# A memristor-based method for discriminating tuberculous and malignant pleural effusions

**DOI:** 10.1016/j.mtbio.2026.103273

**Published:** 2026-05-23

**Authors:** Miaomiao Liu, Jian Wang, Zelin Cao, Bai Sun, Song Ling Wang, Juan Wang, Tao Xin, Ruina Ma, Junxiang Gu, Ping He, Jinbo Zhao, Yu Cui, Teng Wu, Jianqiang Qu, Xiaojun Li, Yandong Nan, Xianxia Yan

**Affiliations:** a Department of Neurosurgery, The Second Affiliated Hospital of Xi'an Jiaotong University, Xi'an, 710004, China; b Department of Respiration, Tangdu Hospital, Fourth Military Medical University, Xi'an, 710038, China; c Department of Thoracic Surgery, Tangdu Hospital, Fourth Military Medical University, Xi'an, 710038, China; d Department of Surgery, 94750th Hospital of Chinese People's Liberation Army, Longyan, 366299, China; e Department of Human Anatomy, Histology and Embryology and K.K. Leung Brain Research Centre, Fourth Military Medical University, Xi'an, 710032, China; f Frontier Institute of Science and Technology (FIST), Xi'an Jiaotong University, Xi'an, 710049, China; g State Key Laboratory of Structural Chemistry, Fujian Institute of Research on the Structure of Matter, Chinese Academy of Sciences, Fuzhou, 350002, China; h Department of Respiratory and Critical Care Medicine, The Second Affiliated Hospital of Xi'an Jiaotong University, Xi'an, 710004, China

**Keywords:** Tuberculous pleural effusion, Malignant pleural effusion, Memristor, Smart medicine

## Abstract

Distinguishing malignant pleural effusion (MPE) from tuberculous pleural effusion (TPE) is important, as the underlying diseases (tuberculosis and advanced malignancies) are major public health concerns. However, an efficient method for differentiating these two effusions is still lacking. In this work, we fabricated an Ag/SiO_2_/Fe_2_O_3_/ITO memristor and demonstrated its ability to reliably distinguish MPE from TPE by exploiting its unique resistive switching behavior. Mechanistic analysis reveals that subtle compositional differences between MPE and TPE can be transduced into distinct, measurable electrical signals. Furthermore, the memristor achieves effective identification of MPE and TPE through characteristic electrochemical responses, most notably polarity-dependent variations in the high-resistance-state/low-resistance-state (R_off_/R_on_) ratio and peak current. This work introduces a memristor-based approach for discriminating MPE and TPE, providing a potential tool for clinical practice.

## Highlights

• It was found that the memristor can effectively distinguish between malignant pleural effusion (MPE) and tuberculous pleural effusion (TPE) through its unique resistance conversion characteristics.
• It has been confirmed that there are inherent compositional differences (like CEA) between MPE and TPE samples that can be effectively converted into measurable electrical signals.
• The memristors can achieve sensitive discrimination between MPE and TPE through their electrochemical response characteristics, particularly the polarity dependent changes in HRS/LRS ratio and peak current.
• This work introduces a memristor-based approach for discriminating MPE and TPE, providing a potential tool for clinical practice.

## Graphical abstract

**Figure:**
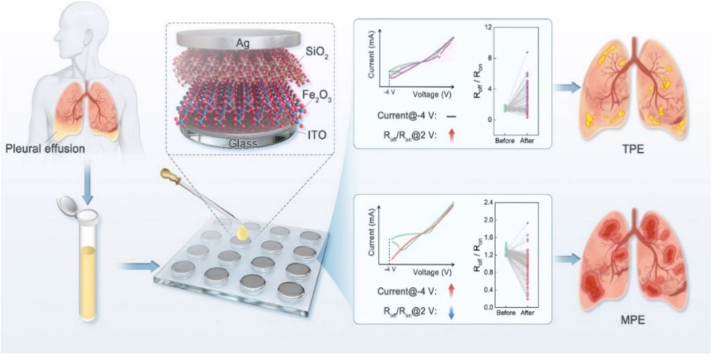

## 1 Introduction

Pleural effusion refers to the abnormal accumulation of fluid within the pleural cavity, which is a common complication of various diseases, including malignancies, tuberculosis infections, cardiovascular diseases, and inflammatory conditions [[1], [2], [3]]. Among these, malignant pleural effusion (MPE) and tuberculous pleural effusion (TPE) are the most prevalent and pose the greatest challenge for clinical differential diagnosis [[4], [5], [6], [7], [8], [9]]. Diagnosis of pleural effusion first relies on imaging studies (e.g., chest radiography, computed tomography, or ultrasonography) to confirm its presence, then followed by thoracentesis to obtain a fluid sample. The type of the effusion is typically characterized through routine fluid analysis, biochemical tests, biomarker assays, and pathological examination [[10], [11], [12], [13]]. However, these current methods face several limitations, including low specificity of biomarkers, time-consuming procedures, and high diagnostic costs [14]. Therefore, there is an urgent need to develop rapid diagnostic and portable preliminary screening approaches.

In recent years, biosensors have emerged as promising tools for point-of-care health monitoring, offering the potential for minimally invasive and real-time detection of disease biomarkers [[15], [16], [17], [18]]. However, most existing sensing systems still rely on silicon-based von Neumann architectures, which face significant computational bottlenecks when processing complex biological signals [19,20]. Moreover, conventional sensors are constrained by their materials, structures and biocompatibility, which in turn limits their clinical application. Given the complexity of bodily fluids, the diversity of detectable analytes, and the heterogeneity of material structures, the integration of novel sensors into biological sample detection has emerged as a major focus of research interest. Memristors, as a new generation of artificial intelligence devices, present a compelling alternative by integrating sensing, memory, and computing functions into a single platform [[20], [21], [22]]. Unlike traditional architectures, memristors enable in-memory computing, which significantly enhances the efficiency for analyzing complex biological signals [23,24]. Another key advantage lies in their material diversity—transition metal oxides, organic polymers, and two-dimensional nanomaterials have all been successfully employed in memristor fabrication [25]. This versatility not only supports low-cost and scalable production but also allows device performance to be tailored for specific detection environments and analyte types. Such properties make memristors ideally suited for detecting complex bodily fluids such as pleural effusions, where multi-analyte profiling and real-time signal interpretation are critical.

In this study, a memristor with an Ag/SiO_2_/Fe_2_O_3_/ITO structure was fabricated using magnetron sputtering for differential detection of pleural effusions. The device exhibited distinct changes in memristive behavior upon exposure to clinically confirmed MPE and TPE. Based on these specific alterations in electrical characteristics, we further assessed the device's capability to discriminate the alterations induced by MPE and TPE on the device's memristive characteristics, we further evaluated its capability to detect suspected pleural effusions. Our findings demonstrate the potential of this memristor-based chip as a rapid and convenient tool for distinguishing between MPE and TPE, offering a novel approach for developing efficient, real-time, accurate, and low-cost detection systems for point-of-care testing of pleural effusion. Importantly, this work is intended as a pioneer study, rather than a formal diagnostic trial. The primary objective is to explore whether memristive devices can generate distinct electrical responses to TPE and MPE, thereby providing a basis for their potential use in clinical discrimination.

## 2 Experimental section

### 2.1 Device preparation

Memristors with an Ag/SiO_2_/Fe_2_O_3_/ITO sandwich structure were fabricated using magnetron sputtering [20,26]. First, the glass substrate with ITO thin film was sequentially ultrasonically cleaned with acetone, ethanol, and deionized water for 15 min each to ensure thorough cleansing. It was then baked on a hot plate at 60 °C for 20 min to remove any moisture adhered to the ITO thin film. Next, Fe_2_O_3_ films were deposited onto the ITO films via magnetron sputtering. The sputtering duration was set to 90 min, with the deposition pressure maintained at 0.5 Pa and the RF power at 60 W. Subsequently, a SiO_2_ film was deposited onto the Fe_2_O_3_ film. The sputtering time was set to 45 min, with a deposition pressure of 0.5 Pa and an RF power of 60 W. Finally, an Ag top electrode was deposited using a dot-patterned metal mask under conditions of 0.8 Pa pressure, 60 W power, and a 15 min sputtering time. The Fe_2_O_3_ and SiO_2_ layers measured to be approximately 243 nm and 45 nm thick, respectively.

### 2.2 Materials characterization

Energy-dispersive X-ray spectroscopy (EDX) and elemental mapping (EDX-mapping) were employed to analyze the composition of the as-deposited Fe_2_O_3_ and SiO_2_ films. The cross-sectional morphology of the Fe_2_O_3_ and SiO_2_ films was examined by scanning electron microscopy (SEM). The crystal structure and phase features of the as-prepared material were characterized via X-ray diffraction (XRD) analysis. All electrical characterization measurements were performed using a Keysight B2901B semiconductor analyzer.

### 2.3 Collection of pleural effusion samples

This study was led by the Second Affiliated Hospital of Xi'an Jiaotong University and the Institute of Frontier Science and Technology, with participation from the Second Affiliated Hospital of Air Force Medical University. All patients were diagnosed upon their first admission and had not received any relevant pharmacological treatment prior to sample collection. The type of pleural effusion was evaluated and determined by senior physicians according to established diagnostic criteria, which integrated thoracoscopy, imaging examinations, pathological analyses, and clinical detection indices (including blood tumor markers, biochemical indices of pleural effusion, and electrolyte levels). Histopathological confirmation served as the gold standard for definitive diagnosis of TPE and MPE. Presumptive TPE or MPE cases were defined as those with negative pathological findings but strong clinical, imaging, or therapeutic evidence supporting the diagnosis. The study was approved by the Ethics Committee of the Second Affiliated Hospital of Xi'an Jiaotong University (Ethics Approval No. 2024251), and written informed consent was obtained from all participants prior to sample collection.

### 2.4 CEA solution preparation

Purified human carcinoembryonic antigen (CEA) was purchased from Shanghai Linc-Bio Science Co., Ltd. And dissolved in saline to prepare solutions at gradient concentrations of 10, 10^2^, 10^3^, and 10^4^ ng/mL. These solutions were used to investigate the concentration-dependent effects of CEA on the electrical characteristics of the memristor device.

### 2.5 Statistical analysis

Data analysis was performed using OriginPro software (OriginLab Corporation). Normality of data distribution was assessed using the Shapiro–Wilk test. Normally distributed continuous variables are expressed as mean ± standard deviation, while non-normally distributed variables are presented as median with interquartile range (IQR). For intra-group comparisons (before *v.* *s.* After treatment), paired Student's t-test was used for normally distributed data, and the Wilcoxon signed-rank test was applied for non-normally distributed data. For inter-group comparisons, independent Student's t-test was used for normally distributed data, and the Mann–Whitney *U* test was used for non-normally distributed data. Statistical significance was defined as *P* < 0.05.

## 3 Results and discussion

### 3.1 Device characterization

**Fig. 1:**
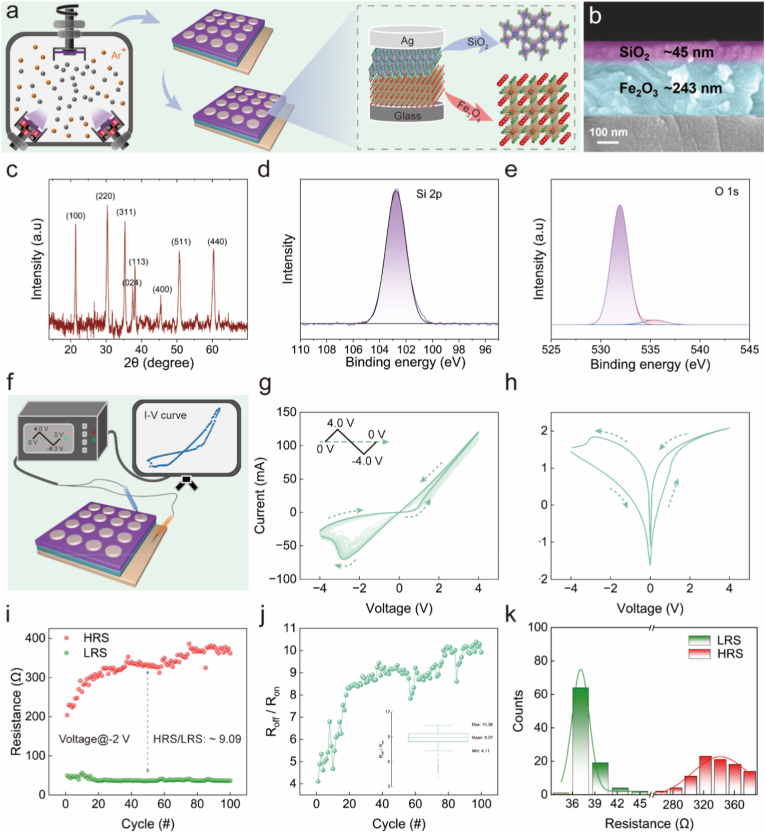
Structure and electrical characteristics of Ag/SiO_2_/Fe_2_O_3_/ITO memristors. (a) Magnetron sputtering fabrication process and sandwich-structured composition of Ag/SiO_2_/Fe_2_O_3_/ITO memristors. (b) Cross-sectional SEM images of SiO_2_ and Fe_2_O_3_ thin films. (c-e) EDX spectra. (f) Schematic diagram of memristor electrical characterization. (g) *I–V* characteristics during 100-cycle scanning within −4.0 V to 4.0 V bias window. (h) Semi–log scale *I–V* curves with arrows indicating voltage sweep direction. (i) Endurance test over 100 switching cycles. (j) Functional relationship between R_off_/R_on_ and switching cycle. (k) Gaussian distribution curves of LRS and HRS with blue/red lines representing Gaussian fitting. (For interpretation of the references to colour in this figure legend, the reader is referred to the Web version of this article.)

In this work, memristive devices with an Ag/SiO_2_/Fe_2_O_3_/ITO sandwich structure were fabricated using magnetron sputtering (Fig. 1a). The cross-sectional SEM image (Fig. 1b) clearly delineates the multilayered structure, showing functional layer thicknesses of approximately 45 nm for SiO_2_ and 243 nm for Fe_2_O_3_. The XRD pattern (Fig. 1c) confirms the coexistence of crystalline Fe_2_O_3_ and ITO phases: characteristic sharp diffraction peaks for Fe_2_O_3_ at 35.58° (311) and 30.20° (220), attesting to its high crystallinity. In contrast, the absence of distinct peaks for SiO_2_ indicates its amorphous nature, functioning as an interfacial buffer layer. X-ray photoelectron spectroscopy (XPS) analysis further corroborates the chemical states: The Si 2p spectrum (Fig. 1d) exhibits a prominent peak at 103.4 eV, corresponding to fully oxidized silicon in SiO_2_. The lack of lower binding energy components confirms the high purity of the silica layer. The O 1s spectrum (Fig. 1e) displays two characteristic peaks at 530.4 eV and 532.3 eV, assigned to Fe_2_O_3_ and SiO_2_ phases, respectively. These binding energies align well with reference values for crystalline Fe_2_O_3_ (529.6–530.2 eV) and thermally grown SiO_2_ (532.3–532.6 eV).

To systematically evaluate the memristive properties of the Ag/SiO_2_/Fe_2_O_3_/ITO device, its *I–V* characteristics were measured over 100 cycles within a voltage sweep range of −4.0 V to 4.0 V (Fig. 1f and g). Arrows in the plot indicate the voltage sweep direction. Throughout this study, a positive voltage denotes a high bias applied to the top Ag electrode, while a negative voltage corresponds to a high bias applied to the bottom ITO electrode. The semi-logarithmic *I–V* curve (Fig. 1h) reveals that the device initially resides in a high-resistance state (HRS). As the sweeping voltage increases, the device switches from HRS to low-resistance state (LRS) at 4.0 V, which is referred to as the SET process. In the negative voltage region, the LRS exhibits fluctuations, showing a narrow switching window. The device undergoes resistance switching (RS) at −4.0 V and maintains a stable HRS, which can be regarded as the RESET process, collectively exhibiting typical bipolar RS behavior. Device performance was further assessed through endurance and retention evaluations. Endurance tests (Fig. 1i) reveal that the LRS has excellent stability, while the HRS shows a certain degree of drift over 100 sweep cycles. Nevertheless, the memristive device maintains a clear distinction between the HRS and LRS, indicating good retention endurance. Meanwhile, the switching ratio (R_off_/R_on_) maintains minor fluctuations within a range of 4.11–10.38 over 100 sweep cycles (Fig. 1j), demonstrating overall good endurance and operational robustness. Statistical analysis of the HRS and LRS distribution measured at a read voltage of −2.0 V follows a Gaussian profile (Fig. 1k), further supporting the uniformity and repeatability of the device.

These results align with previously reported multilayer memristors based on atomic-layer-deposited iron oxide, which exhibit bipolar switching behavior with robust endurance and retention characteristics [27]. The *I–V* characteristics and resistive switching properties of such memristors have been employed in our two latest studies to discriminate between different types of brain tumor cells and to detect the occurrence of intracerebral hemorrhage [28,29]. These further supports the reliability of selecting such devices as testing platforms.

### 3.2 Saline control and device-to-device reproducibility

**Fig. 2:**
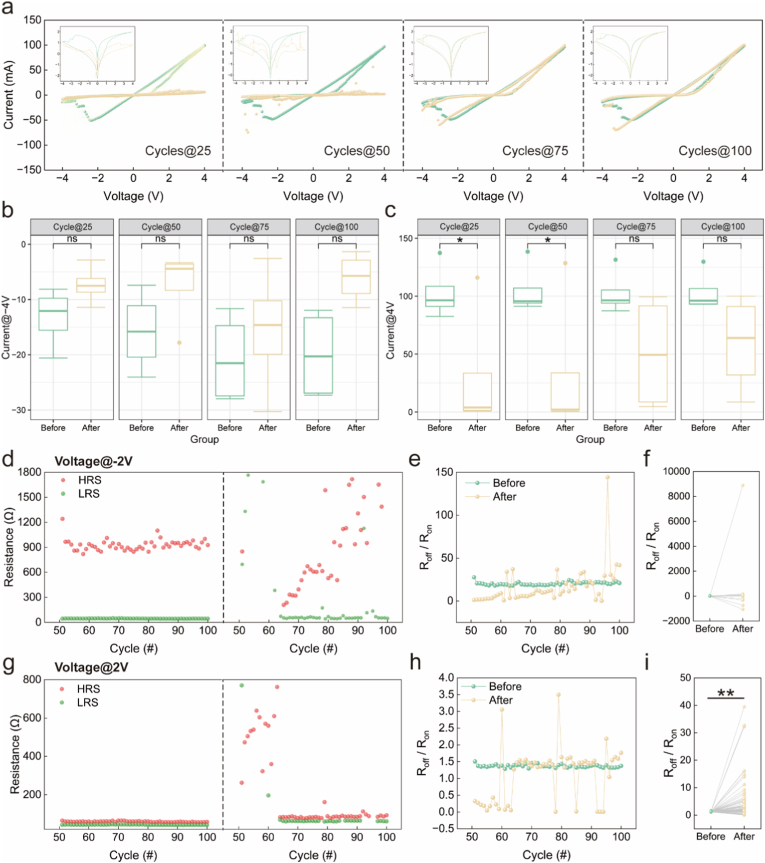
Influence of saline on electrical characteristics of Ag/SiO_2_/Fe_2_O_3_/ITO memristors. (a) *I–V* curves of the memristors before and after saline application, with the 25th, 50th, 75th, and 100th cycles highlighted. (b, c) Current values recorded at −4.0 V and 4.0 V during the 25th, 50th, 75th, and 100th cycles (n = 4). (d, g) Retention properties before and after saline application at reading voltages of −2.0 V and 2.0 V, device impedance stabilizes after 50 cycles following saline application. (e, f) And (h, i) Switching characteristics from the 50th to 100th cycles before and after saline application at reading voltages of −2.0 V and 2.0 V *P < 0.05, **P < 0.01, After *v.s.* Before.

First, saline was tested as a negative control to determine whether the physiological solution itself would induce any non-specific electrical response in the memristor. We characterized the memristive behavior through 100 consecutive voltage sweeping cycles. Representative *I–V* curves from the 25th, 50th, 75th, and 100th cycles were highlighted for comparison between pre- and post-saline application (Fig. 2a). The pristine device exhibited typical bipolar non-volatile RS behavior, consistent with the device characteristics shown in Fig. 1g. After saline application, the *I–V* curves showed increased fluctuation during the initial 50 cycles, then remained stable. However, statistical analysis of multiple devices revealed no significant difference in the current values at either −4.0 V (Fig. 2b, n = 4) or 4.0V (Fig. 2c, n = 4). Endurance analysis was performed using the stabilized *I–V* curves (cycles 50-100). The pristine device exhibited negligible deterioration in both LRS and HRS at read voltages of −2.0 V (Fig. 2d, left) and 2.0 V (Fig. 2g, left), maintaining a stable R_off_/R_on_ ratios disparity (Fig. 2e and h, before). After saline application, the HRS and LRS exhibited notable fluctuations at a voltage of −2.0 V (Fig. 2d, right), whereas they remained relatively stable at 2.0 V (Fig. 2g, right). Statistical analysis showed that, despite the observed fluctuations, the R_off_/R_on_ ratios at −2.0 V after saline application were not significantly different from those of the pristine device (Fig. 2e and f). In contrast, at 2.0 V, the R_off_/R_on_ ratios showed significant increase (Fig. 2h and i). These findings indicate that while saline transiently affects the I–V curves and the HRS and LRS, the device performance becomes relatively stable after 50 cycles and remains comparable to that of the pristine device. Therefore, the peak current at ±4.0 V and the switching ratios at ±2.0 V were selected as key parameters for evaluating different types of pleural effusion.

To further evaluate the device-to-device reproducibility and the stability of the key parameters, we characterized 20 independent memristor devices. The *I–V* hysteresis loops of the 20 devices exhibited highly consistent profiles (Fig. S1). The distributions of current values at ±4.0 V (Fig. S2) and the R_off_/R_on_ ratios at ±2.0 V (Fig. S3) showed no obvious dispersion, confirming excellent inter-device uniformity and satisfactory fabrication reproducibility. These results support the reliability of using the peak current at −4.0 V and the switching ratio at ±2.0 V as stable electrical signatures for discriminating different types of pleural effusion.

### 3.3 Electrical response of Ag/SiO_2_/Fe_2_O_3_/ITO memristor exposed to TPE

**Fig. 3:**
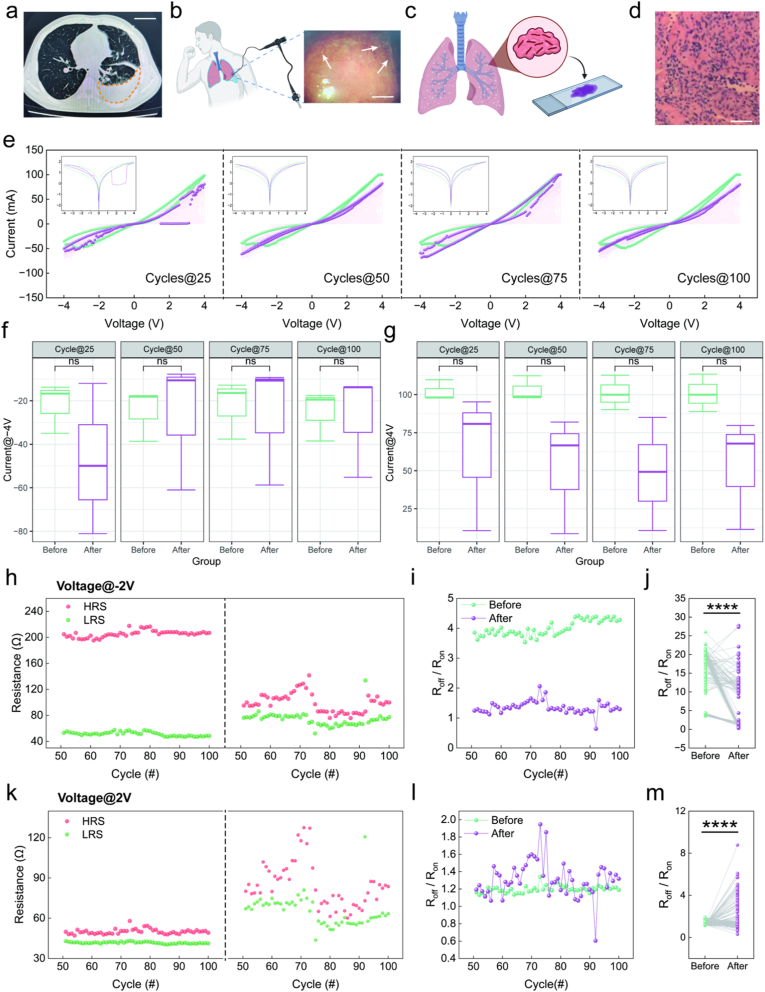
Influence of TPE on electrical characteristics of Ag/SiO_2_/Fe_2_O_3_/ITO memristors. (a) A chest CT scan from a diagnosed TPE patient. (b)Thoracoscopic view showing caseous necrotic tissue caused by *Mycobacterium tuberculosis* infection (indicated by arrows). (c, d) Histopathological biopsy procedure and staining results showing granulomatous inflammation with lymphocytic infiltration. (e) *I–V* curves of the memristors before (green) and after (purple) TPE application, with the 25th, 50th, 75th, and 100th cycles highlighted. (f, g) Current values recorded at −4.0 V and 4.0 V during the 25th, 50th, 75th, and 100th cycles (n = 9). (h, i) Retention properties before and after TPE application at reading voltages of −2.0 V and 2.0 V, device impedance stabilizes after ∼50 cycles following TPE application. (j, k) Switching characteristics from the 50th to 100th cycles before and after TPE application at reading voltages of −2.0 V and 2.0 V. Bar in a, b and d is 50 mm, 5 mm, and 50 μm respectively. ****P < 0.0001, After *v.s.* Before. (For interpretation of the references to colour in this figure legend, the reader is referred to the Web version of this article.)

The TPE was characterized by substantial pleural effusion on CT (Fig. 3a), thoracoscopic observation of typical tuberculous miliary nodules (Fig. 3b), and pathological confirmation of chronic granulomatous inflammation caused by tuberculosis (Fig. 3c and d). Following drop-casting of TPE onto the device, we characterized its memristive behavior through 100 consecutive voltage sweeping cycles. Representative *I–V* curves from the 25th, 50th, 75th, and 100th cycles were highlighted for comparison between pre- and post-TPE application (Fig. 3e). The pristine device exhibited typical bipolar non-volatile RS behavior, consistent with the device characteristics shown in Fig. 1g. After TPE application, the *I–V* curves showed increased fluctuation during the initial 50 cycles, then remained stable. The change in peak current at 4.0 V was more pronounced than at −4.0 V. However, statistical analysis of multiple devices revealed no significant difference in the current values at either −4.0 V (Fig. 3f, n = 9) or 4 V (Fig. 3g, n = 9). Endurance analysis was performed using the stabilized *I–V* curves (cycles 50-100). The pristine device exhibited negligible deterioration in both LRS and HRS at read voltages of −2.0 V and 2.0 V, maintaining a stable R_off_/R_on_ ratios disparity (Fig. 3h and k, left). After TPE application, the device's HRS and LRS were significantly altered in a voltage-polarity-dependent manner. At a read voltage of −2.0 V, TPE treatment markedly decreased the HRS and increased the LRS (Fig. 3h, right), leading to a significant decrease in the R_off_/R_on_ ratios (Fig. 3i and j). Conversely, at a read voltage of 2.0 V, the increase in HRS was significantly greater than that in LRS after TPE application (Fig. 3k, right), resulting in an increase of the switching ratio (Fig. 3l and m). These results demonstrate that TPE treatment induces voltage-polarity-dependent modulation of RS characteristics, with consistent enhancement under positive bias and suppression under negative bias.

### 3.4 Electrical response of Ag/SiO_2_/Fe_2_O_3_/ITO memristor exposed to MPE

**Fig. 4:**
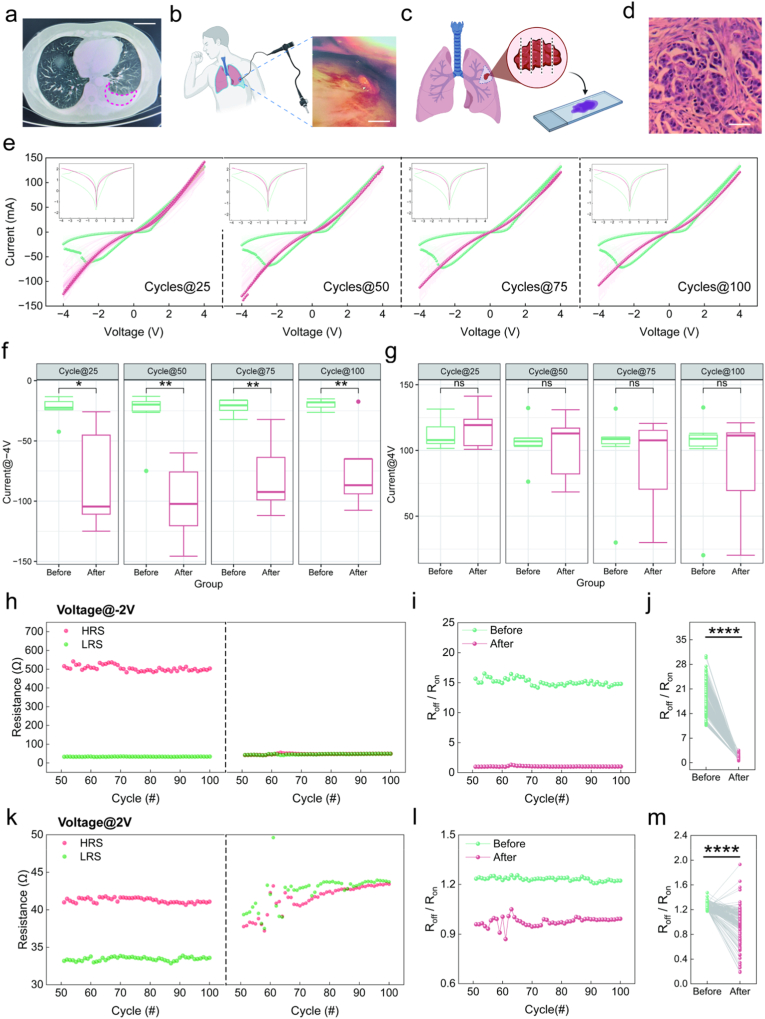
Influence of MPE on electrical characteristics of Ag/SiO_2_/Fe_2_O_3_/ITO memristors. (a) A chest CT scan from a diagnosed MPE patient. (b) Thoracoscopic view showing tumor nodules. (c, d) Histopathological biopsy procedure and staining results showing tumor cells. (e) *I–V* curves of the memristors before and after MPE application, with the 25th, 50th, 75th, and 100th cycles highlighted. (f, g) Current values recorded at −4.0 V and 4.0 V during the 25th, 50th, 75th, and 100th cycles (n = 12). (h and k) Retention properties before and after MPE application at reading voltages of −2.0 V and 2.0 V. (i, j and l, m) Switching characteristics from the 50th to 100th cycles before and after MPE application at reading voltages of −2.0 V and 2.0 V. Bar in a, b and d is 50 mm, 5 mm, and 50 μm respectively. *P < 0.05, **P < 0.01, ****P < 0.0001, After *v.s.* Before.

The MPE sample was characterized by substantial pleural effusion on CT (Fig. 4a), thoracoscopic identification of a tumor nodule (Fig. 4b), and a histopathological diagnosis of metastatic lung carcinoma (Fig. 4c and d). Following MPE application, we characterized the memristive behavior through 100 consecutive voltage sweeping cycles. Representative *I–V* curves (Fig. 4e) from the 25th, 50th, 75th, and 100th cycles were highlighted for comparison with their pristine states. Over 100 sweep cycles, the current at −4.0 V demonstrated progressive enhancement, whereas that at 4.0 V showed no significant change. Statistical analysis confirmed that the current at −4.0 V was significantly increased at all measured cycles (Fig. 4f and g, P < 0.05 at cycle 25; *P* < 0.01 at cycles 50, 75, 100; n = 12). Endurance analysis revealed pronounced MPE effects: The pristine devices, in contrast, maintained a stable HRS/LRS disparity under both bias conditions (Fig. 4h and k, left). At a read voltage of −2.0 V, MPE application significantly decreased HRS without markedly altering LRS (Fig. 4h, right), leading to a reduced switching ratio (Fig. 4i and j). Similarly, at a read voltage of 2.0 V, MPE also significantly decreased HRS and slightly increased LRS (Fig. 4k, right), which likewise resulted in a significant reduction of the switching ratio (Fig. 4l and m). These results indicate that MPE treatment suppresses the switching ratio under both positive and negative voltage biases, while significantly enhancing the current at the negative peak voltages.

### 3.5 Electrical response of Ag/SiO_2_/Fe_2_O_3_/ITO memristor exposed to IPE

To assess whether the device can differentiate TPE and MPE from non-malignant, non-tuberculous inflammatory pleural effusions (IPE), we tested clinically confirmed IPE samples as an additional control. For IPE, representative *I–V* curves from the 25th, 50th, 75th, and 100th cycles were highlighted for comparison between pre- and post-IPE application (Fig. S4a). The pristine device exhibited typical bipolar non-volatile RS behavior, consistent with the device characteristics shown in Fig. 1g. After IPE application, the *I–V* curves showed no significant difference in the current values at either −4.0 V (Fig. S4b, n = 3) or 4.0 V (Fig. S4c, n = 3). Endurance analysis was performed using the stabilized *I–V* curves (cycles 50-100). The pristine device exhibited negligible deterioration in both LRS and HRS at read voltages of −2.0 V (Fig. S4d, left) and 2.0 V (Fig. S4g, left), maintaining a stable R_off_/R_on_ ratios disparity (Fig. S4e and h). After IPE application, the HRS and LRS of the device exhibited significant fluctuations at a voltage of −2.0 V (Fig. S4d, right) and 2.0 V (Fig. S4g, right), and the switching ratio of the device showed a significant decrease after IPE treatment (Figs. S4e, f, h, i). These results indicate that IPE does not alter the *I–V* curve shape of the Ag/SiO_2_/Fe_2_O_3_/ITO device but reduces the ratio of HRS to LRS at both −2.0 V and 2.0 V.

### 3.6 Summary of electrical signatures

In summary, as shown in Table 1, the memristive responses of the four tested groups (saline, IPE, TPE, and MPE) exhibited distinct electrical signatures. Saline only increased the switching ratio at 2.0 V. IPE and MPE both reduced the switching ratio at −2.0 V and 2.0 V, but MPE uniquely increased the peak current at −4.0 V, whereas IPE did not. TPE showed a polarity-dependent modulation of the switching ratio: decreased at −2.0 V but increased at 2.0 V, with no change in peak current. These characteristic patterns demonstrate that the Ag/SiO_2_/Fe_2_O_3_/ITO memristor can effectively discriminate among saline, IPE, TPE, and MPE based on the combination of peak current at −4.0 V and switching ratio at ±2.0 V.

**Table 1:** The electrical signatures of saline, IPE, TPE, and MPE.

Category	Current @ −4.0V	Current @ 4.0V	R_off_/ R_on_ @ −2.0V	R_off_/R_on_ @ 2.0V
Saline	**—**	**—**	**—**	**↑**
IPE	**—**	**—**	**↓**	**↓**
TPE	**—**	**—**	**↓**	**↑**
MPE	**↑**	**—**	**↓**	**↓**

### 3.7 Mechanistic investigations

A total of 24 pleural effusion samples were included in this study, comprising 12 cases clinically diagnosed as MPE, 9 cases as TPE, 3 cases as IPE. Among the confirmed diagnostic groups, the mean age of patients with MPE was 64.75 ± 3.98 years, that of patients with TPE was 61.89 ± 4.49 years, and with IPE was 69.00 ± 3.06 years, no statistically significant difference was found among the three groups (*F* = 0.352, *P* = 0.707), supporting the validity of intergroup comparisons.

To further explore the potential molecular basis underlying the distinct memristive responses, we measured a panel of biomarkers (Table S1) and biochemical and ion concentrations (Table S2) in the pleural effusion samples. No significant differences were observed among the MPE, TPE, and IPE groups in lactate dehydrogenase (LDH), total protein (TP), or electrolyte levels (K^+^, Na^+^, Ca^2+^, Cl^−^) (all *P* > 0.05). Although an increasing trend in adenosine deaminase (ADA) was observed in the clinically confirmed TPE group, no statistically significant difference was found among the groups (*P* = 0.086). Among all measured tumor markers, only carcinoembryonic antigen (CEA) in MPE was significantly higher than those in TPE and IPE (*P* = 0.014). These results suggest that the observed memristive signatures are not attributable to general physical properties such as ionic strength or protein concentration, but rather may be driven by specific molecular components such as CEA.

**Fig. 5:**
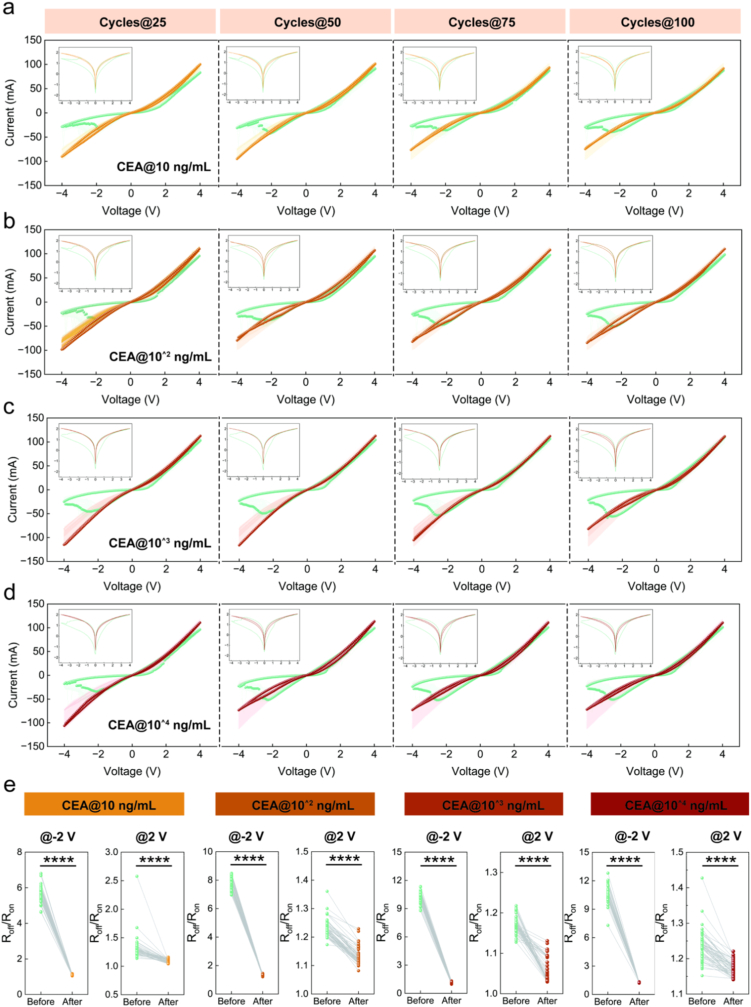
Influence of CEA on electrical characteristics of Ag/SiO_2_/Fe_2_O_3_/ITO memristors. (a, d) *I–V* curves of the memristors before and after different concentrations of CEA application (range from 10 ng/mL to 10^4^ ng/mL), with the 25th, 50th, 75th, and 100th cycles highlighted. (e) Switching characteristics from the 50th to 100th cycles before and after CEA application (range from 10 ng/mL to 10^4^ ng/mL) at reading voltages of −2.0 V and 2.0 V ****P < 0.0001, After *v.s.* Before.

Subsequently, we applied CEA solutions at various concentrations onto the surface of the Ag/SiO_2_/Fe_2_O_3_/ITO device to observe their effects on its electrical characteristics. Interestingly, within the concentration range from 10 ng/mL to 10^4^ ng/mL, the influence of CEA on the *I–V* curve of the device exhibited a trend consistent with that observed for MPE (Fig. 5a−d). Analysis of the switching ratio revealed that at both −2.0 V and 2.0 V, all tested concentrations of CEA significantly reduced this ratio (Fig. 5e, *P* < 0.0001). These results indicate that CEA may contribute to the distinctive memristive response observed for MPE.

To further verify the physical interaction between biomolecules and the device surface, we performed ex-situ XRD analysis on memristor devices after exposure to TPE and MPE samples (Fig. S5). No obvious shift in the position or intensity of the characteristic diffraction peaks was observed, confirming that the device substrate remained structurally intact without corrosion or damage caused by the biological samples. Notably, the Ag (111) peak intensity was significantly higher and sharper after TPE treatment, whereas it was markedly reduced and broadened after MPE treatment. This difference directly reflects the specific interaction of Ag with biomolecules in the two types of pleural effusion. For MPE, negatively charged macromolecules may bind to Ag^+^ ions under positive bias, hindering the formation of intact Ag conductive filaments, which explains the decreased Ag peak intensity and the increased LRS resistance.

### 3.8 Application to clinically suspected cases

**Fig. 6:**
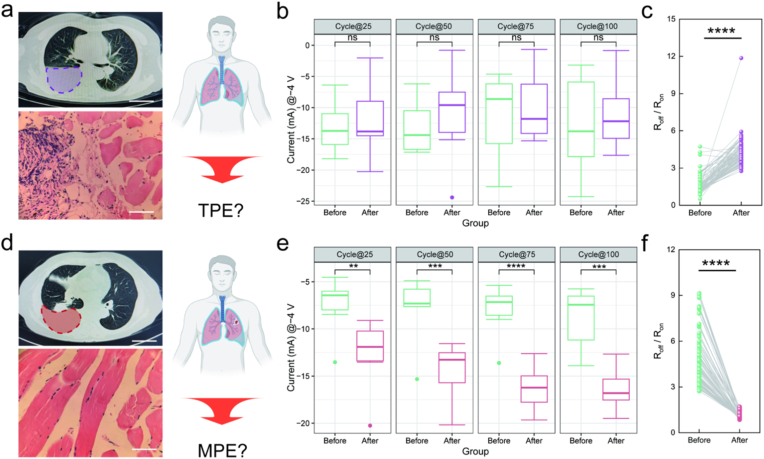
Memristive Signature-Based Discrimination for Pathologically Uncertain TPE *v.s.* MPE. (a) Chest CT scan and histopathological results from a suspected MPE patient. (b) Current values recorded at −4.0 V during the 25th, 50th, 75th, and 100th cycles before and after suspected MPE application. (c) Switching characteristics from the 50th to 100th cycles before and after suspected MPE application at reading voltages of 2.0 V. (d) Chest CT scan and histopathological results from a suspected TPE patient. (e) Current values recorded at −4.0 V during the 25th, 50th, 75th, and 100th cycles before and after suspected TPE application. (f) Switching characteristics from the 50th to 100th cycles before and after suspected TPE application at reading voltages of 2.0 V. Bar in (a) and (f) is 50 mm and 50 μm respectively. **P < 0.01, ***P < 0.001, ****P < 0.0001, After *v.s.* Before.

The Ag/SiO_2_/Fe_2_O_3_/ITO device exhibits distinct memristive responses to confirmed IPE, MPE and TPE samples. As a complementary demonstration of the device's discriminative capability, we further investigated its utility in distinguishing clinically suspected cases. We evaluated one suspected MPE case and one suspected TPE case. Both patients had inconclusive pathological biopsy results (Fig. 6a and d), potentially due to sampling non-lesional tissue during thoracoscopy. However, comprehensive clinical assessment (tumor marker profiles, T-SPOT results, and treatment responses) supported MPE and TPE diagnoses, respectively.

The Ag/SiO_2_/Fe_2_O_3_/ITO device demonstrates potential distinguish capability between suspected MPE and TPE. Following the application of suspected TPE, the electrical characteristics of the device exhibit a trend consistent with that observed after the application of clinically confirmed TPE, specifically characterized by fluctuations in current at −4.0 V without statistically significant differences (Fig. 6b), and an increase in the R_off_/R_on_ ratios at 2.0 V (Fig. 6c, *P* < 0.0001). In contrast, after the application of suspected MPE, the electrical characteristics of the device are also highly consistent with the trend observed after the application of clinically confirmed MPE, specifically manifested as an increase in current at −4.0 V (Fig. 6e, *P* < 0.0001) and a decrease in the R_off_/R_on_ ratios at 2.0 V (Fig. 6f, *P* < 0.0001).

These results demonstrate that the Ag/SiO_2_/Fe_2_O_3_/ITO memristor could effectively identifies the suspected MPE and TPE samples. Crucially, the changes of R_off_/R_on_ ratios trends for the suspected cases align closely with those of confirmed pleural effusions, demonstrating its robust ability to differentiate between MPE and TPE. This highlights the device's potential as a promising platform for rapid and definitive determination of pleural effusion.

## 4 Conclusions

In summary, the Ag/SiO_2_/Fe_2_O_3_/ITO memristor can effectively discriminate among TPE, MPE, and IPE based on distinct electrical signatures: the peak current at −4.0 V and the R_off_/R_on_ ratios at ± 2.0 V. The discriminative capability was validated using confirmed cases, suspected cases, and control groups (saline and IPE). Mechanistic studies further identified CEA as a molecular contributor to the MPE-specific signature. This study is exploratory and proof-of-concept in nature. Formal diagnostic trials with larger sample sizes, blinding, and comparison with a gold standard would be required in the future.

## CRediT authorship contribution statement

**Miaomiao Liu:** Conceptualization, Data curation, Writing – original draft. **Jian Wang:** Conceptualization, Data curation, Writing – original draft. **Zelin Cao:** Formal analysis, Software. **Bai Sun:** Supervision, Writing – review & editing. **Song Ling Wang:** Formal analysis, Validation. **Juan Wang:** Resources. **Tao Xin:** Visualization. **Ruina Ma:** Investigation. **Junxiang Gu:** Validation. **Ping He:** Conceptualization. **Jinbo Zhao:** Methodology. **Yu Cui:** Validation. **Teng Wu:** Resources. **Jianqiang Qu:** Formal analysis. **Xiaojun Li:** Validation. **Yandong Nan:** Supervision. **Xianxia Yan:** Supervision, Writing – review & editing.

## Declaration of competing interest

The authors declare that they have no known competing financial interests or personal relationships that could have appeared to influence the work reported in this paper.

## Data Availability

Data will be made available on request.
