# Supplementary material for: A memristor-based method for discriminating tuberculous and malignant pleural effusions
Source: Mater Today Bio. 2026 May 23;38:103273. doi: 10.1016/j.mtbio.2026.103273 (PMC13226219; doi:10.1016/j.mtbio.2026.103273)
Supplement: Multimedia component 1 [file mmc1.docx]

**Supplementary Material**

**A memristor-based method for discriminating tuberculous and malignant pleural effusions**

Miaomiao Liu ^a,b,1^, Jian Wang ^c,d,e,1^, Zelin Cao ^f,1^, Bai Sun ^a,f,^*, Song Ling Wang ^g^, Juan Wang ^b^, Tao Xin ^b^, Ruina Ma ^b^, Junxiang Gu ^a,e^, Ping He ^h^, Jinbo Zhao ^c^, Yu Cui ^f^, Teng Wu ^a,f^, Jianqiang Qu ^a^, Xiaojun Li ^f^, Yandong Nan ^b,^*, Xianxia Yan ^a,^*

^a^ *Department of Neurosurgery, The Second Affiliated Hospital of Xi’an Jiaotong University, Xi’an 710004, China*

^b^ *Department of Respiration, Tangdu Hospital, Fourth Military Medical University, Xi’an 710038, China*

^c^ *Department of Thoracic Surgery, Tangdu Hospital, Fourth Military Medical University, Xi’an 710038, China*

^d^ *Department of Surgery, 94750^th^ Hospital of Chinese People’s Liberation Army, Longyan, 366299, China*

^e^ *Department of Human Anatomy, Histology and Embryology and K.K. Leung Brain Research Centre, Fourth Military Medical University, Xi’an 710032, China*

^f^ *Frontier Institute of Science and Technology (FIST), Xi’an Jiaotong University, Xi’an 710049, China*

^g^ *State Key Laboratory of Structural Chemistry, Fujian Institute of Research on the Structure of Matter, Chinese Academy of Sciences, Fuzhou 350002, China*

^h^ *Department of Respiratory and Critical Care Medicine, The Second Affiliated Hospital of Xi’an Jiaotong University, Xi’an 710004, China*

^1^*These authors contributed equally to this work.*

**Email:* [*baisun@xjtu.edu.cn*](mailto:baisun@xjtu.edu.cn) *(B.S.);* [*13709205538@163.com*](mailto:13709205538@163.com) *(Y.N.);* [*yanxx07@xjtu.edu.cn*](mailto:yanxx07@xjtu.edu.cn) *(X.Y.).*


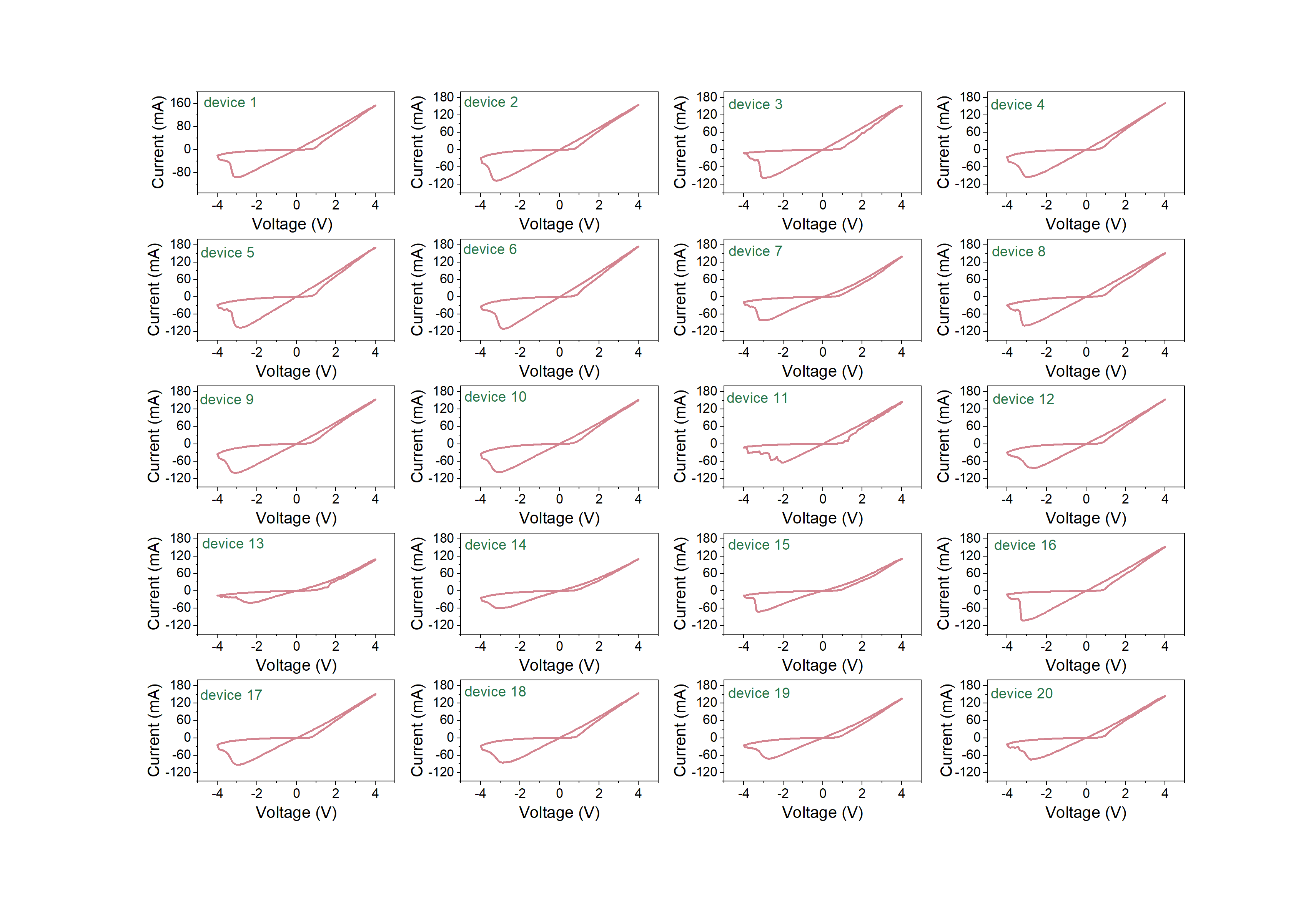


**Figure S1**. The *I-V* curves of the 20 tested memristors.


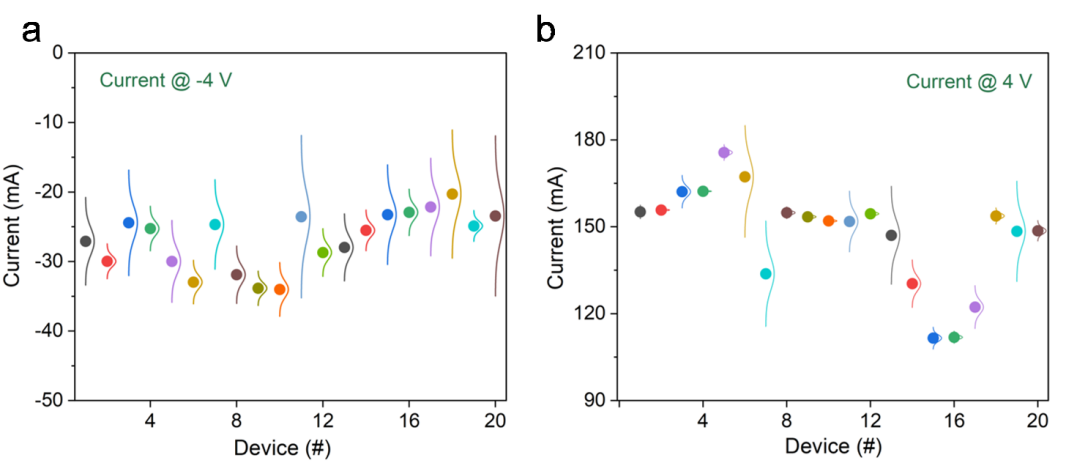


**Figure S2**. Statistical plot of currents for the 20 tested memristors at **a)** a read voltage of −4.0 V. **b)** a read voltage of 4.0 V.


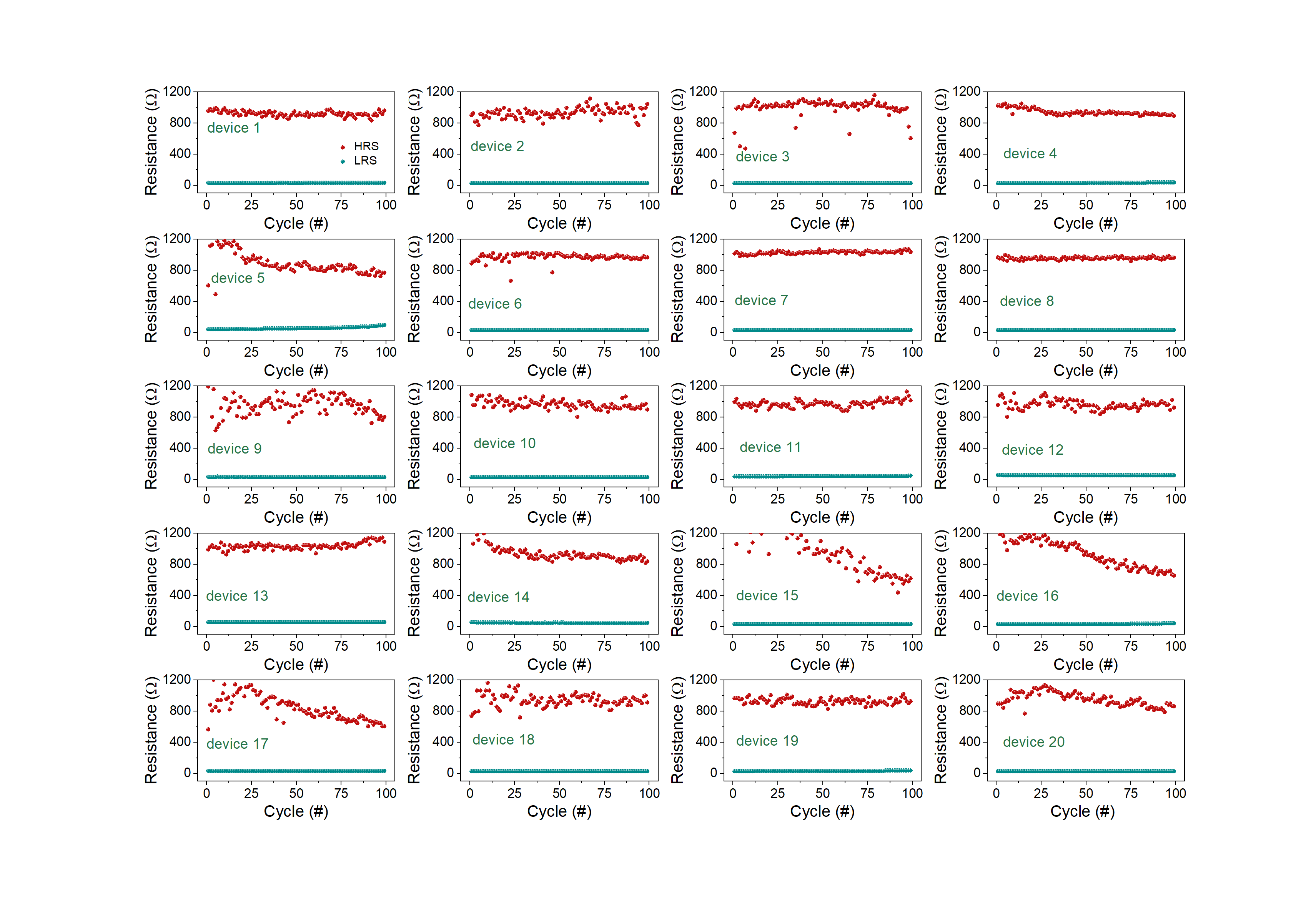


**Figure S3**. The HRS/LRS ratios of the 20 tested memristors.


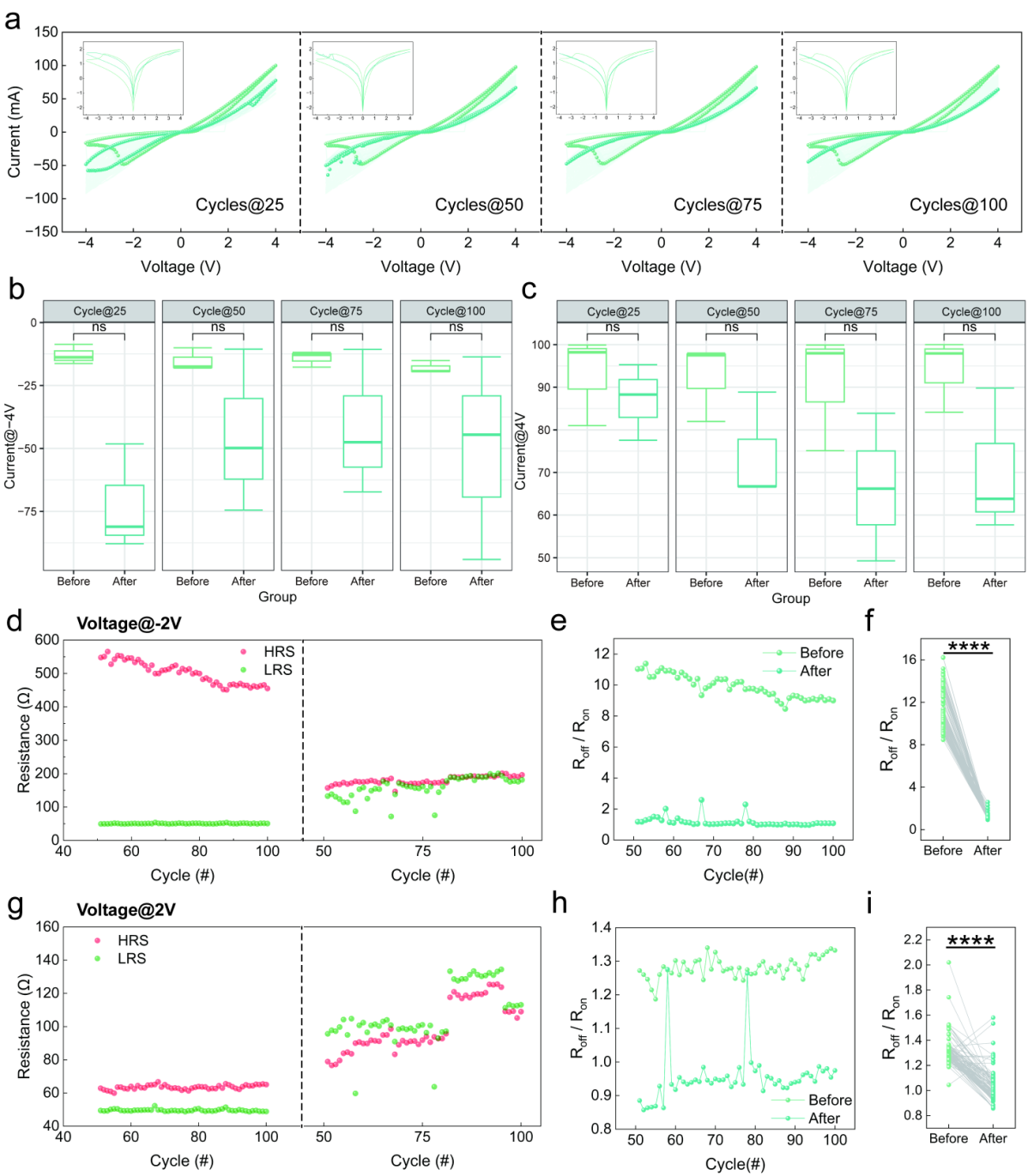


**Figure S4.** Influence of IPE on electrical characteristics of Ag/SiO_2_/Fe_2_O_3_/ITO memristors. **a)** *I-V* curves of the memristors before and after IPE application, with the 25^th^, 50^th^, 75^th^, and 100^th^ cycles highlighted. **b, c)** Current values recorded at −4.0 V and 4.0 V during the 25^th^, 50^th^, 75^th^, and 100^th^ cycles (n = 3). **d, g)** Retention properties before and after IPE application at reading voltages of −2 V and 2 V, device impedance stabilizes after ~50 cycles following IPE application. **e-f, h-i)** Switching characteristics from the 50^th^ to 100^th^ cycles before and after IPE application at reading voltages of −2.0 V and 2.0 V.


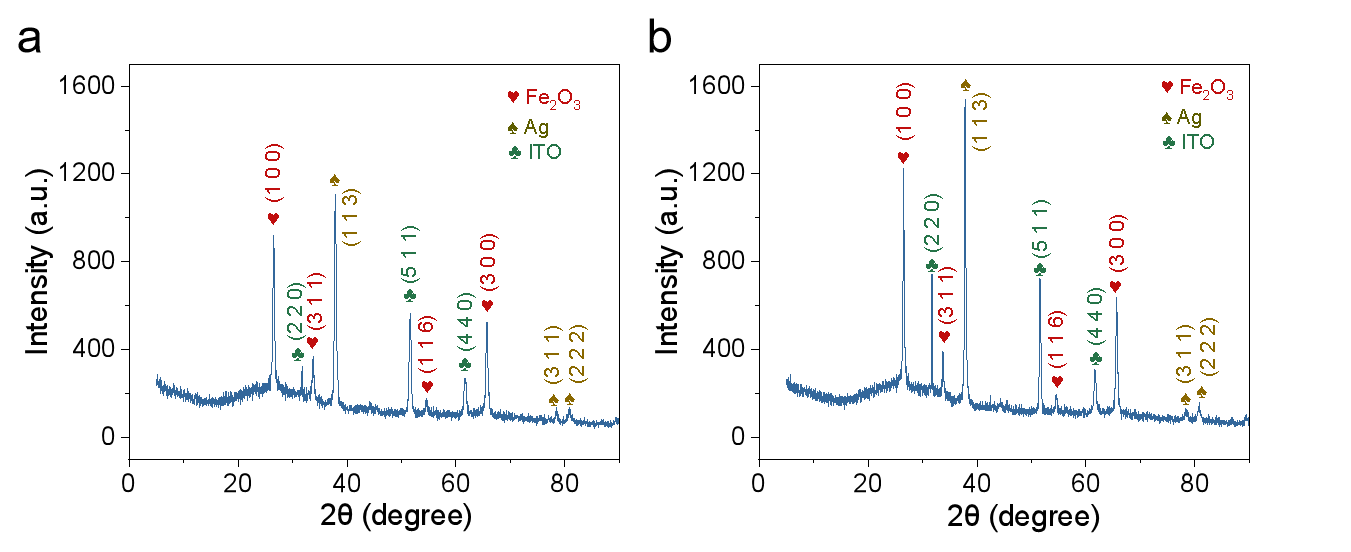


**Figure S5**. XRD patterns of the characterized memristor devices. **a)** TPE treated group. **b)** MPE treated group.

**Table S1** Comparison of biomarkers levels in blood and pleural effusion.

| Category | Sample | CEA  (ng/mL) | Ferritin  (ug/L) | NSE  (ng/mL) | CA199  (ng/mL) | LDH  (U/L) | ADA  (U/L) |
| --- | --- | --- | --- | --- | --- | --- | --- |
| MPE | 12 | 84.89±24.88 | 489.33±119.86 | 17.57±2.62 | 6.75±1.12 | 291.08±63.47 | 14.25±3.32 |
| TPE | 9 | 2.03±0.47 | 329.78±63.72 | 15.37±2.05 | 12.70±6.25 | 275.11±35.28 | 20.84±6.95 |
| IPE | 3 | 2.08±1.05 | 288.33±110.53 | 12.9±1.35 | 1.73±0.35 | 125±16.77 | 11±1.15 |
| F value |  | 5.291 | 0.822 | 0.530 | 1.174 | 1.144 | 2.771 |
| *P* value |  | 0.014 | 0.453 | 0.596 | 0.329 | 0.338 | 0.086 |

**Table S2** Comparison of biochemical index levels in pleural effusion.

| Category | Sample | TP  (g/L) | Glu  (mmol/L) | K^+^  (mmol/L) | Na^+^  (g/L) | Ca^2+^  (mmol/L) | Cl^-^  (mmol/L) |
| --- | --- | --- | --- | --- | --- | --- | --- |
| MPE | 12 | 36.97±2.01 | 6.54±0.68 | 3.69±0.12 | 140.32±1.04 | 2.15±0.04 | 107.45±1.10 |
| TPE | 9 | 43.29±3.57 | 6.41±0.62 | 3.69±0.16 | 139.84±0.40 | 2.17±0.04 | 106.57±1.53 |
| IPE | 3 | 42.47±0.69 | 12.41±2.51 | 4.25±0.18 | 138.93±1.54 | 2.23±0.01 | 111.1±0.49 |
| F value |  | 1.634 | 7.586 | 2.257 | 0.302 | 0.470 | 1.487 |
| *P* value |  | 0.219 | 0.003 | 0.129 | 0.743 | 0.632 | 0.249 |
